# Ultrasound-Guided Transversus Abdominis Plane Block versus Continuous Wound Infusion for Post-Caesarean Analgesia: A Randomized Trial

**DOI:** 10.1371/journal.pone.0103971

**Published:** 2014-08-05

**Authors:** Michel Chandon, Agnès Bonnet, Yannick Burg, Carole Barnichon, Véronique DesMesnards-Smaja, Brigitte Sitbon, Christine Foiret, Jean-François Dreyfus, Jamil Rahmani, Pierre-Antoine Laloë, Marc Fischler, Morgan Le Guen

**Affiliations:** 1 Department of Anesthesiology, Hôpital Foch, Suresnes, France; 2 Department of Anesthesiology, Institut Hospitalier Franco-Britannique, Levallois-Perret, France; 3 Department of Clinical Research and Innovation, Hôpital Foch, Suresnes, France; 4 Department of Health Education, Bradford Teaching Hospitals, Leeds, United Kingdom; Karolinska Institutet, Sweden

## Abstract

**Objective:**

To compare the analgesic effect of ultrasound-guided Transversus Abdominis Plane (TAP) block versus Continuous Wound Infusion (CWI) with levobupivacaine after caesarean delivery.

**Methods:**

We recruited parturients undergoing elective caesareans for this multicenter study. Following written informed consent, they received a spinal anaesthetic without intrathecal morphine for their caesarean section. The postoperative analgesia was randomized to either a bilateral ultrasound guided TAP block (levobupivicaine = 150 mg) or a CWI through an elastomeric pump for 48 hours (levobupivacaine = 150 mg the first day and 12.5 mg/h thereafter). Every woman received regular analgesics along with oral morphine if required. The primary outcome was comparison of the 48-hour area under the curve (AUC) pain scores. Secondary outcomes included morphine consumption, adverse events, and persistent pain one month postoperatively.

**Results:**

Recruitment of 120 women was planned but the study was prematurely terminated due to the occurrence of generalized seizures in one patient of the TAP group. By then, 36 patients with TAP and 29 with CWI had completed the study. AUC of pain at rest and during mobilization were not significantly different: 50 [22.5–80] in TAP versus 50 [27.5–130] in CWI (*P* = 0.4) and 190 [130–240] versus 160 [112.5–247.5] (*P* = 0.5), respectively. Morphine consumption (0 [0–20] mg in the TAP group and 10 [0–32.5] mg in the CWI group (*P* = 0.09)) and persistent pain at one month were similar in both groups (respectively 29.6% and 26.6% (P = 0.73)).

**Conclusion:**

In cases of morphine-free spinal anesthesia for cesarean delivery, no difference between TAP block and CWI for postoperative analgesia was suggested. TAP block may induce seizures in this specific context. Consequently, such a technique after a caesarean section cannot be recommended.

**Trial Registration:**

ClinicalTrials.gov NCT01151943

## Introduction

Caesarean delivery is one of the most commonly performed surgical procedures and postoperative pain is a great concern for women [1]. Its severity may impair early postoperative maternal rehabilitation and recovery [2]. Opioids and non-steroidal anti-inflammatory drugs, as components of a multimodal analgesic regimen, achieve effective analgesia. However both systemic and neuraxial opioid administration are associated with frequent dose-dependent adverse effects including nausea, vomiting, pruritus, sedation, respiratory depression, hyperalgesia [3] and are transferred across the placenta to the fetus. Thus, alternatives such as peripheral nerve block or wound infiltration have been suggested especially for cases where general anesthesia is indicated or where intrathecal opioids are contra-indicated. Seeing that a single dose of local anesthetic wound infiltration only offers short-term analgesia in the postoperative period [4], a regional block or a continuous local anesthetic technique may provide better analgesia. Two recent meta-analyses have shown the efficacy of single shot bilateral Transversus Abdominis Plane (TAP) block [5], [6] after caesarean section while another demonstrated the usefulness of local anesthetic agent with Continuous Wound Infiltration (CWI) during a few days [7]. The former has been improved by ultrasound guidance [8] and the latter by placement of a multi−orifice catheter below the fascia [9]. To date, no prospective trial has directly compared the analgesic efficacy of these two techniques.

We therefore conducted a study to compare continuous delivery of local anesthetic using a wound catheter and ultrasound-guided bilateral TAP block after elective caesarean delivery during the first 48 postoperative hours.

## Results

**Figure 1:**
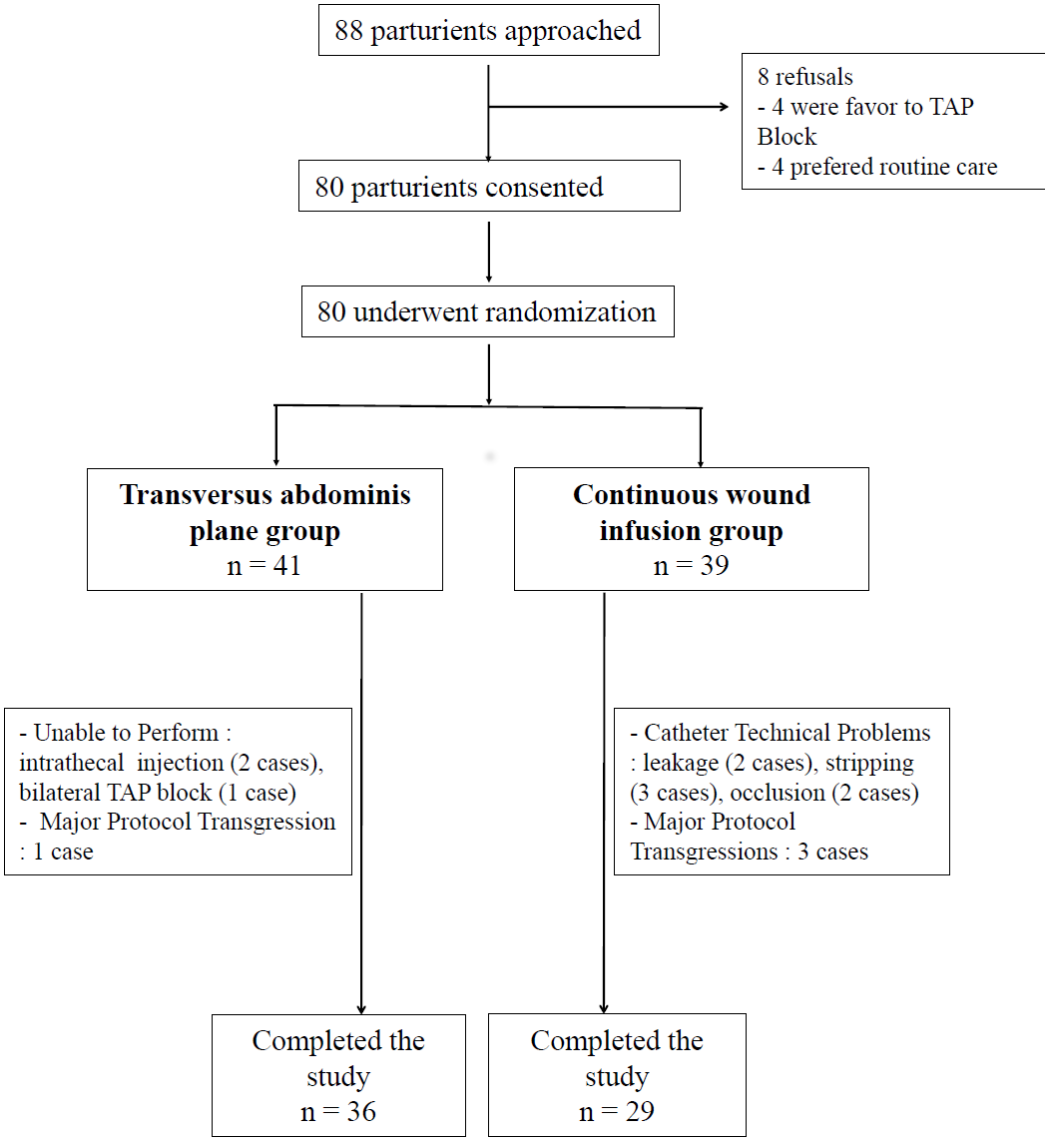
Consort trial diagram.

The study began September 2010 and was prematurely stopped in July 2011 due to the occurrence of generalized convulsions a few minutes after a TAP block. This event required transient mechanical ventilation and was successfully treated using an intravenous lipid emulsion infusion. It has been considered to be the consequence of a partial systemic absorption of local anesthetic. An independent pharmaco-vigilance committee has recommended stopping the trial because this event if expected as every regional technique occurred with a high rate (1/40) that was higher than expected. Moreover, physiological changes of pregnancy may favor its occurrence and further research is required. Consequently, only 80 subjects were enrolled in the study, 39 were randomized to the CWI group and 41 to the TAP group. Ten patients did not complete the study in the CWI group and 5 in the TAP group as seen in the Consort diagram (Figure 1).

**Figure 2:**
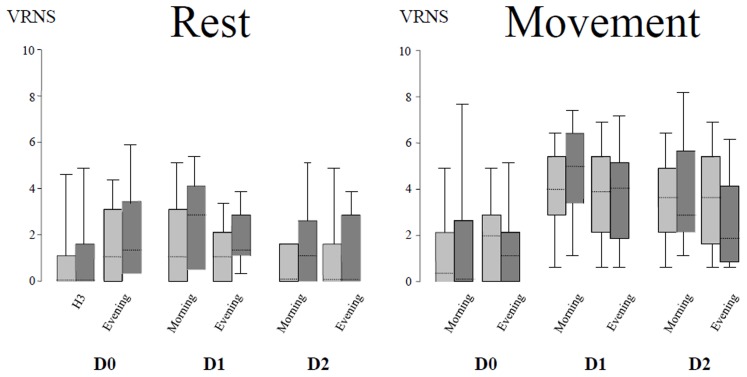
Pain scores at D0 (3rd postoperative hour and evening), D1 (morning and evening) and D2 (morning and evening). Results are represented by box plot. The horizontal solid line gives the median value and the upper and lower limit give the interquartile range. At last, the 5 and 95th percentiles correspond to the limit of the whiskers. Left panel: pain scores at rest. Right panel: pain scores during mobilization. VRNS: verbal response numerical scale pain score.

Demographic characteristics, as well as intraoperative parameters were similar (Table 1). The Intention to Treat analysis showed that pain at rest was low in the postoperative period in both groups with a median AUC of pain scores during 48 hours at 50 [22.5–80] in the TAP group and at 50 [27.5–130] in the CWI group (*P* = 0.3). During mobilization, pain was higher but similar in both groups with an AUC at 190 [130–240] in the TAP group and at 160 [105–262] in the CWI group (P>0.9). The per-protocol analysis has given similar results with an AUC at rest at 70 [30–110] and 50 [22.5–120] (*P* = 0.4) and during mobilization at 190 [130–240] and 160 [112.5–247.5] respectively (*P* = 0.5) (Figure 2). The mixed model for repeated measures showed a non-significant group effect both for pain scores at rest (*P* = 0.5) and pain scores with movement (*P* = 0.5). In both cases the time factor was significant (*P*_rest_ = 0.03, *P*_movement_ = 0.00002) showing a significant change in the perception of pain along time. The time × group interaction is not significant (*P*_rest_ = 0.6, *P*_movement_>0.9) as we were unable to show a different time profile of effect for the two procedures.

**Table 1:** Characteristics of study patients, presented as mean [1^st^ and 3^rd^ interquartile] and as numbers (%).

	Transversus abdominis plane block Group		Continuous wound infusion Group	
	ITT	PP	ITT	PP
	n = 41	n = 36	n = 39	n = 29
Age (yr)	34 (30–37)	33 (30–37)	32 (30–37)	32 (30–38)
Height (cm)	164 (160–168)	165 (160–169)	165 (161–170)	164 (160–170)
Weight (kg)	72 (65–81)	74 (68–82)	73 (68–81)	74 (68–81)
Surgical time (min)	40 (34–48)	40 (35–50)	45 (37–50)	45 (40–60)
Surgical difficulties (%)	7 (17.0)	7 (19.4)	11 (28.2)	9 (31.0)
Previous abdominal surgery (%)	19 (46.3)	19 (52.7)	25 (64.1)	17 (58.6)

ITT: *Intent to Treat* Analysis (all included patients); PP: *Per Protocol* Analysis (patients who completed the study protocol).

No woman required rescue intravenous morphine administration in the post-anesthesia care unit. Oral morphine was not required by the third postoperative hour in the TAP Block group but was required in 2 cases in the CWI group (*P = *0.21). Median oral morphine consumption was not significantly different between the groups: 0 [0–20] mg in the TAP group and 10 [0–32.5] mg in the CWI group (*P* = 0.09), with a large inter-individual variability. Seventeen patients of 36 (47%) in the TAP group compared to 16 patients out of 29 (55%) in the CWI group took oral morphine during the first 60 postoperative hours (*P* = 0.7).

Patients experienced nausea and vomiting more frequently in the TAP group (*P* = 0.03). No difference was reported concerning other opioid related adverse effects (pruritus and sedation). A wound complications were seen in 2 cases from the CWI group (*P* = 0.19). Patient satisfaction with pain relief did not significantly differ between groups (Table 2).

**Table 2:** Side effects and Satisfaction, presented as numbers (%).

		Transversus Abdominis Plane block Group	Continuous wound infusion Group	*P* value
		n = 36	n = 29	
Nausea and vomiting		6 (16.7)	0 (0)	0.03
Pruritus		7 (19.4)	6 (20.7)	>0.9
Gastrointestinal function reestablishment at 48 h				
	Flatus	33 (91.7)	24 (82.7)	>0.9
	Faeces	12 (33.3)	8 (27.6)	>0.9
Sedation*		5 (13.9)	4 (13.8)	>0.9
Surgical wound complication**		0 (0)	2 (6.9)	0.18
Satisfaction Scores				>0.9
1 or 2		35 (97.2)	27 (93.1)	
3 or 4		1 (2.8)	1 (3.4)***	

*cases with mild to moderate sedation,

**slight oozing from the scar and scar haematoma,

***one missing data.

Median postoperative length of stay was similar in both groups (6 [5]–[7] days) (*P* = 0.9). Twenty seven patients in the TAP group (66%) and 19 patients (49%) in the CWI group responded to the postoperative interview. They reported similar rates of persistent wound pain and of neuropathic pain (Table 3).

**Table 3:** Main results obtained from the a follow-up postal standardized questionnaire, presented as numbers (%).

	Transversus Abdominis Plane block Group	Continuous wound infusion Group	*P* value
	n = 27	n = 19	
Incidence of pain in the scar area (VRNS)	8 (29.6)	4 (26.6)	0.73
Neuropathic pain*	2 (7.4)	4 (26.6)	0.21

VRNS: Verbal Response Numerical Scale,

*defined as score of 4 or above on the DN4 questionnaire [11].

## Discussion

The occurrence of generalized seizures occurring a few minutes after an ultrasound guided TAP block led to the premature study termination. Consequently this randomized trial was underpowered to detect any significant difference in term of analgesia between continuous wound infiltration and TAP block in cases of scheduled caesarean delivery under spinal anesthesia.

Local anesthetic agent wound infiltration has been proposed after various surgical procedures and a meta-analysis by Gupta et al. demonstrated that wound catheters provided no significant analgesia at rest or on activity, except in patients undergoing gynecological and obstetric surgery [13]. Another meta-analysis by Bamigboye and Hofmeyr confirmed the interest of this technique after caesarean section [7].

Efficacy of a wound infiltration technique using a local anesthetic/non-steroidal anti-inflammatory drug mixture [14] is increased when the catheter tip is placed below the fascia [9]. The safety of wound infiltration was demonstrated by Gupta et al. in their meta-analysis: low incidence of side effects and above all no statistically significant differences for wound infection, wound erythema, and hematoma when compared with control patients, except for a lower risk for wound breakdown in patients receiving a local anesthetic [13]. In our study, technical problems were frequently encountered - 7 cases (17.9%) - with inadvertent stripping of the catheter in the operating theatre or during the transfer from operating table to bed, leakage, and occlusion despite cautious during patient mobilization. Even though they claimed global satisfaction with the technique, several women stated that it was uncomfortable to have a wound catheter and an elastomeric pump for forty hours post-operatively; however, we did not systematically study this point.

On the other hand, TAP block techniques are gaining wide acceptance, especially with ultrasound guidance [8]. Results from two meta-analyses are contradictory. The first one concluded that there is only limited evidence to suggest that usage of perioperative TAP block reduces opioid consumption and pain scores after abdominal surgery when compared with no intervention or placebo [15]. A more recent meta-analysis concluded that TAP block is safe, reduces postoperative morphine requirements, nausea and vomiting and possibly the severity of pain after abdominal surgery [16]. If there is some doubt regarding its indication after abdominal surgery, conversely, two meta-analyses have shown efficacy of bilateral TAP block after caesarean section [5], [6]. These meta-analyses did not report major adverse events. We are only aware of one previous published case of convulsions which occurred 3 hours after a TAP block in a patient that had had a laparoscopy-assisted myomectomy [17].

This study showed no significant difference between local anesthetic agent wound infiltration and TAP block especially in terms of postoperative analgesia but it must be outlined that there was a wide inter-individual variability in the dose of oral morphine showing that both techniques have variable efficacy. Adverse effects and pain persisting at one month concerned 25 to 30% of the patients, a value higher than that reported in the literature (14–18%), were similar in both groups [2], [18]. Previously described risk factors such as pre-existing pain, acute postoperative pain, a young age and general anesthesia cannot explain these results. The short follow-up time (one month versus 3 months in literature) and modalities of study and interview (prospective series with DN4 interview) may have affected our findings. Finally, designing this study with a morphine-free spinal anesthetic, most likely had an effect on immediate to intermediate pain scores.

The limitations of the study are as follows. We did not include the whole sample of 120 patients as initially planned by the statistical analysis because of the occurrence of a major undesirable event in the TAP group. Furthermore, several patients were excluded from analysis as described above. It can be postulated that the study is underpowered; however it is unlikely that a significant difference between groups could have been found with a complete set of patients. A second major limitation is due to the fact that our study was not blinded and that patients systematically received paracetamol, ketoprofen, and nefopam. This could reduce the difference between the studied loco-regional techniques but represents our routine postoperative care. Conversely, our use of intrathecal bupivacaine with sufentanil instead of a longer acting opioid such as morphine avoids a supplementary confounding factor in our analysis of the analgesic effect and side effects. This is important as a recent study showed that TAP block is associated with greater supplemental morphine requirements and higher pain scores than intrathecal morphine after caesarean section [19].

In conclusion, our randomized trial was prematurely ended do to the occurrence of a severe adverse event following a TAP block demonstrating that local anesthetic toxicity can occur even with continuous ultrasound guidance. Despite the fact that our study is consequently underpowered, a difference in term of analgesic efficacy between TAP block and wound infiltration seems improbable regarding our results. As a consequence, use of TAP block after caesarean cannot be recommended before further pharmacological studies on the relationship between adverse effect to local anesthetic and pregnancy.

## Methods

This multicenter randomized clinical open trial with parallel arms was approved by an Ethic Committee (Comité de Protection des Personnes, Hôpital A. Paré, Boulogne Billancourt, France) and by the French national regulatory office (Agence Française de Sécurité Sanitaire des Produits de Santé). The trial was registered on ClinicalTrials.gov as NCT01151943. The protocol for this trial and supporting CONSORT checklist are available as supporting information; see Checklist S1 and Protocol S1.

Parturients were eligible if they were scheduled for an elective caesarean delivery at term with a low transverse incision (Joel-Cohen incision) under spinal anesthesia. Other criteria of inclusion were American Society of Anesthesiologists physical status 1 or 2 and a singleton fetus >37 weeks of gestation. Non-inclusion criteria were women in active labour, age<19 or >40 years old, height<155 cm, weight<50 kg or a Body Mass Index>35 kg/m^2^ and women with contra-indication to any of the study protocol analgesics (paracetamol, ketoprofen, nefopam and levobupivacaine). Exclusion criteria were conversion from spinal to general anesthesia owing to inadequate intraoperative analgesia, failure of the neuraxial technique, failure to site the multi-hole catheter (CWI group) and failure to visualize the intrafascial space (TAP group).

Some weeks (from 4 to 10) before the caesarean delivery, a pre-anesthetic visit was performed during which oral and written information about spinal anesthesia and the study protocol were given. Written informed consent was then obtained either the day before or on the day of surgery. Upon admission to the operative room, every parturient received a standard monitoring including electrocardiogram, non-invasive arterial pressure, and pulse oximetry. At this moment, randomization was performed using a computer-generated set of scratch cards with blocks of 6 and a ratio 1∶1 for each arm, and patients were assigned to one of the two groups for postoperative analgesia TAP or CWI. Intrathecal anesthesia was standardized with a dural puncture at level L3–L4 and administration of 0.5% hyperbaric bupivacaine (8 mg if height<1.60 m, 10 mg otherwise) plus sufentanil 5 µg. After the block was established, the caesarean section was performed. In the TAP group, a trained anesthesiologist performed US-guided TAP block just after completion of surgery [10] injecting 20 mL of levobupivacaine 0.375% (150 mg) bilaterally. TAP block is a regional anesthetic technique that blocks the abdominal wall neural afferents (T6-L1) into the neurofascial plane between the internal oblique and transversus abdominis muscles. A bilateral injection is required after cesarean section. Ultrasound allows real-time identification the three layers of abdominal muscles: external oblique, internal oblique, and transversus abdominis and visualization the spread of the local anesthetic. The quality of the detachment of the two muscle fascia spreading was recorded by the investigator but no attempt by checking sensory level was made to verify that the TAP block was working. In the CWI group, prior to skin closure, the surgeon inserted under aseptic conditions a multihole catheter (Painfusor Baxter 15 cm), previously tested with a bolus of saline to check catheter patency. The catheter was placed below the fascia between the unclosed parietal peritoneum and the underside of the transversalis fascia before its closure, along the full length of the wound [9]. The catheter was then connected to an elastomeric pump (Infusor LV5 Baxter) containing 250 mg levobupivacaine in 200 mL solution set to deliver 5 mL per hour, i.e. 150 mg the first 24 postoperative hours. For ethical reasons, patients and investigators were not blinded. Oral paracetamol (1 gram), ketoprofen (50 mg) and nefopam (20 mg), four times a day completed the multimodal analgesia plan.

In the post-anesthesia care unit, intravenous morphine was titrated if required (verbal response numerical scale (VRNS) pain score ≥4 on a scale ranging from 0 = no pain to 10 = unbearable. VRNS pain scores ≥4 were managed with oral morphine (morphine sulfate, 10 mg per tablet; Mundipharma, Paris, France) in the post-natal ward. Ondansetron and symptomatic treatment of vomiting was available if required. For the purpose of the study, nurses, not involved in the study, from the mobile pain unit assessed patients 3 hours post-cesarean delivery, in the evening (between 18∶00 and 22∶00, allowing for a time interval of 6 to 12 hours since the first assessment) of the operative day (D0), in the morning (around 9∶00) and the evening (between 18∶00 and 20∶00) of the first (D1) and second (D2) postoperative days. They collected also every adverse event of the techniques for safety concern. During the assessment, the women were asked to rate pain with VRNS at rest and when moving (from supine to sitting position in bed), to report any pruritus and any nausea or vomiting (absent or present). The level of sedation (1 = awake and alert, 2 = mild sedation or asleep but easily roused, 3 = moderate sedation, and 4 = unable to rouse) was also reported. For the purpose of statistical analysis, sedation was present if a woman had a score of two or more. Satisfaction with pain control was recorded immediately before the patient left hospital with a VRNS (1 = very satisfied, 2 = satisfied, 3 = dissatisfied, 4 = very dissatisfied). Gastrointestinal function on the second postoperative day (flatus and faeces), and length of stay at hospital were also recorded.

One month post−delivery, the women were interviewed by phone by an investigator blinded to the patient's group assignment. Women were asked if there was any pain in the scar area. In case of a positive answer, the incidence of neuropathic pain was evaluated using the DN4 questionnaire, such a diagnosis being made if the score was equal or greater than 4 on a 0–10 scale [11]. A consultation for chronic postoperative pain could be organized 3 months after the delivery.

### Statistical analysis

The primary outcome was pain over the 48 h postoperative period, i.e. period of local anesthetic in the CWI group, at rest and during mobilization using the area under the curves (AUC) of pain scores. This method allows a better precision in analgesic efficacy than individual pain score reports. AUC was calculated by the trapezoidal method [12] considering that the first measurement was made 3 hours after surgery, the second one 9 hours after the first one and the following ones every 12 hours thereafter. Secondary outcomes included the time to the first rescue dose of morphine, the total morphine requirements during the first postoperative 48 hour period, adverse events, patients’ satisfaction, length of hospital stay, and prevalence of neuropathic postoperative pain.

The study was designed to detect a between means ratio of 1.25 in either direction for the AUC, with 85% power allowing for an attrition rate of 10%. The coefficient of variation of the means was hypothesized to be 0.4. With a bilateral significance level of 0.05, the sample size was calculated at 60 patients per group. The Intention to Treat population included all patients that were randomized to the study whatever the treatment they actually received and the Per-Protocol population includes subjects who completed the follow-up in each group.

Since the AUC pain scores were skewed and not normalized by a logarithmic transformation, nonparametric tests (Mann-Whitney) were used for group comparisons. Categorical variables, expressed as numbers with frequencies (%) were compared using Chi−square and Fisher's exact tests as appropriate. Continuous variables and pain scores are reported as median with [interquartile range]. A mixed model for repeated measures with two primary factors (pain control method: CWI or TAP and time) and an interaction term was fitted. The Akaike criterion was used to select the most appropriate model. P values <0.05 were considered statistically significant. No interim analysis was planned. Statistical analysis was performed using NCSS (Versions 8 and 9, Kaysville, USA).

## Supporting Information

Checklist S1 CONSORT Checklist.
(DOC)

Protocol S1 Trial Protocol.
(DOC)
