# Supplementary material for: Ultrasound-Guided Transversus Abdominis Plane Block versus Continuous Wound Infusion for Post-Caesarean Analgesia: A Randomized Trial
Source: PLoS One. 2014 Aug 5;9(8):e103971. doi: 10.1371/journal.pone.0103971 (PMC4122362; doi:10.1371/journal.pone.0103971)
Supplement: Protocol S1 — Trial Protocol. (DOC) [file pone.0103971.s002.doc]

**Protocol**

**1. GENERAL INFORMATION**

**1.1. Titles**

- Full Title of the research: Analgesia after cesarean section by continuous wound infiltration or bilateral Transversus Abdominis Planus block in single injection (study of equivalence)

- Short Title: Cesar-Pain

- Version Number: version 3 dated May 3, 2011

- Protocol code number assigned by the promoter: 2010/15

- EUDRACT Number: 2010-019662-88

**1.2. Study Sponsor**

Hôpital Foch, 92150 Suresnes

**1.3. Name and quality of the person authorized to sign the protocol and possible changes on behalf of the promoter**

Professor Marc Fischler, anesthesia department, Hôpital Foch (research coordinator)

**1.4. Name and quality of the main investigator**

- Principal Investigator and Coordinator: Professor Marc Fischler, Head of anesthesia department, Hopital Foch (tel 0146252442, fax 0146252088, e-mail: m.fischler@hopital-foch.org)

- Investigators and place of research: Drs Veronique des Mesnard-Smaja, Christine Foiret, Michel Chandon, Isabelle Aimé, Camille Cornet, Janet Siwik, Kerstin Sievert, Benoit Vandenbunder, Olivier Pruszkowski, Morgan Le Guen, Bertrand Rives, Julie Bresson, Lea Ley, Séverine Rosant, Agnès Declerck, Julie Beranger, anesthesia department, Hopital Foch

**Co-investigators:**

- Investigators and places of research: Drs Agnes Bonnet Bourichon, Imad Ben Henda Arwa, Carole Barnichon, anesthesia department, Institut Hospitalier Franco-Britannique

- Dr Dominique Collard and Dr. Yves Cukierman, anesthesia department and intensive care unit, Hôpital Diaconesses - La Croix Saint Simon

- Dr Thibaut Rackelboom,  Monique Berl and Gregory Dubar, Anesthesia department and Intensive Care unit, Hôpital Cochin, AP-HP

**2. SCIENTIFIC JUSTIFICATION AND GENERAL DESCRIPTION OF RESEARCH**

**2.1. General Concepts**

 Cesarean delivery is in constant increase and the postoperative pain is classified as strong (1). The maximal intensity occurs during the first 48 hours following surgery and decreases gradually over two to three days. This acute pain is characterized by a dual component: a somatic one linked to the surgical incision and a visceral one linked to uterine contractions.

To date, several analgesic strategies are proposed, based on a multimodal approach combining morphine (systemic or perimedullar) or local anesthetics with non-opioid analgesics such as paracetamol, nonsteroidal anti-inflammatory drugs, tramadol or nefopam.

2.1.1. Morphine

Many modes of administration exist such as the intrathecal route, intra-venous injection or oral administration.

- The intrathecal route: intrathecal doses of morphine ranging from 0.075 mg to 0.5 mg provide an analgesic effect of quality over a period covering up to 24 h post-surgery. The data of two randomized studies (2, 3) and a meta-analysis (4) show a ceiling effect from doses of 0.075 to 0.1 mg of morphine. Despite this dose, adverse effects remain frequent. Pruritus appears as the most frequent and the most troublesome undesirable effect after intrathecal administration (5). Pruritus was observed in one patient for 2.6 (IC 95 %, 2.1-3.3) patients treated with a dose of 0.05-0.25 mg of intrathecal morphine. For nausea the risk is increased by 6.3 fold (CI 95 %, 4.2-12.5) and 10.1 fold (CI 95 %, 5.7- 41.0) for vomiting (4). In addition, the incidence of pruritus, nausea and vomiting is dose-dependent. Clinically significant respiratory depression is a sporadic occurrence but severely morbid. It may be delayed by 6-10 h or even more, due to cephalic migration via the cerebrospinal fluid. Obesity is the main risk factor for alveolar hypoventilation (6). Most cesarean deliveries are performed under spinal anesthesia for safety reasons (mother and fetus) and it is usual to administer 0.1 mg of morphine to provide a correct analgesia covering the first 24 postoperative hours. Despite a theoretical and large beneficial profile, patient satisfaction is limited by the high rate of residual pain and especially by the undesirable effects that limit post-operative comfort.

- Intra-venous administration: analgesia, controlled by the patient for morphine is widely prescribed as the postoperative technique and especially after cesarean section. It is compatible with breastfeeding due to negligible residual concentrations of morphine or metabolites in the breast milk (7). But its side effects are also frequent, as for intrathecal administration of morphine: pruritus, nausea and vomiting but also sedation. Moreover, the self-administration pump and its tubing can restrict the patient (for mobility and caring for her new-born baby)... The major interest of patient-controlled analgesia is to avoid the risk of respiratory depression. This administration of iv morphine is systematically associated with non opioid analgesics to reduce the doses of morphine and therefore the adverse effects.

2.1.2.  Non-morphine analgesics

It is recommended to associate multimodal analgesics: paracetamol, tramadol, nefopam, and especially non-steroidal anti-inflammatory drugs. Non-steroidal anti-inflammatory drugs are effective on the pain associated with the incision and on the visceral component of pain (5, 8). Nevertheless, non-steroidal anti-inflammatory agents alone are insufficient to control pain after cesarean section (9). Numerous studies have demonstrated that their administration in association with opioids in systemic or in perimedullar spaces, significantly improves quality of analgesia and reduces the adverse effects of morphine (10-13).  The limits of the use of non-steroidal anti-inflammatory agents are essentially their gastrointestinal adverse effects and their inhibitory effect on platelet function. On this last point, the selective inhibitors of cyclo-oxygenase type 2 seem less deleterious. However, these inhibitors pass into breast milk and the experience of these agents in lactating women is very limited at present. The non-steroidal anti-inflammatory drugs such as ketorolac or ibuprofen are secreted into breast milk, but at low concentrations, and are generally regarded as without risk in the event of breast feeding (5). Finally, rare cases of uterine atony after use of ketorolac or diclofenac postpartum have been reported without a clear demonstrated relationship  (5).

2.1.3. Local anesthetics

Two techniques allow the postoperative administration of local anesthetics: continuous wound infiltration with local anesthetics or troncular and regional blocks of the abdominal wall (ilio-inguinal block and more recently transverse abdomen block).

2.1.4. Synthesis

The analgesia after cesarean section should meet several aims. It must be effective, with minimal pain to allow quick recovery and the ability to walk and to care for the new-born baby. For the same reasons, it must cause the least possible side effects, and it must not interfere with maternal activity. Morphine, whatever the route of administration, is effective but causes some side effects and regional analgesia has the same disadvantages.

The techniques for the administration of isolated local anesthetics are still not widespread and are promising with a favorable ratio "analgesic benefit - undesirable effects".

**2.2. NAME AND DESCRIPTION OF THE INVESTIGATIONAL MEDICINAL PRODUCT**

The investigational drug is levobupivacaine (Chirocaine) administered either by sub-cutaneous wound infiltration or through the TAP block (block of bilateral transverse of the abdomen in a single injection).

**2.3. SUMMARY OF THE RESULTS OF THE CLINICAL TRIALS AVAILABLE AND RELEVANT IN THE CONTEXT OF THE BIOMEDICAL RESEARCH CONCERNED**

Several tests have focused on the techniques which allow a loco-regional application of local anesthetics:

- Continuous wound infiltration with local anesthetics (14-16). Sometimes it is associated with non-steroidal anti-inflammatory agents (17),

- The regional blocks of the abdominal wall (ilioinguinal block and more recently transverse abdomen block– TAP block) (18, 19).

A recent meta-analysis including 13 studies has compared effectiveness of continuous wound infiltration with a catheter and local infiltration of the scar. They have showed that the latter in single injection decreased the consumption of morphine and the pain scores significantly more than continuous wound infiltration and this was effective up to 48 h (20).

**2.4. SUMMARY OF THE BENEFITS, IF ANY, AND OF THE FORESEEABLE RISKS KNOWN TO THE PEOPLE AGREEING TO TAKE PART IN THE RESEARCH**

**2.4.1. Benefit for patients**

The expected benefit is an improvement of postoperative analgesia allowing an early rehabilitation.

**2.4.2. Risks caused by participation in the research**

The induced risks are the techniques of analgesia proposed:

- Risks related to the levobupivacaine: the regimens proposed are in conformity with the usual recommendations (Annex 1: Summary of characteristics of Chirocaine 1.25 mg/ml; Annex 2: Summary of characteristics of Chirocaine 2.5 mg/ml; Annex 3: Summary of characteristics of Chirocaine 5 mg/ml).

- Risks of analgesics (paracetamol, ketoprofen, morphine): the contra-indications will be respected and the regimens proposed are in conformity with the usual recommendations. A particular point is the use of ketoprofen among women who breastfeed. The RCP of the ketoprofen mentioned "The NSAIDs passing in breast milk, as a precaution, it is appropriate to avoid the administration to women who are breast feeding." However, the ARCT (Reference Center on the teratogenic agents of the Hospital Armand Trousseau, - www.lecrat.org) mentioned "the quantity of ketoprofen ingested via milk is very low: the child receives less than 1% of the maternal dose (in mg/kg). No particular event has been reported among children, breastfed infants, of mothers receiving ketoprofen. In the light of these elements, the use of ketoprofen is possible by a woman who is breast-feeding. "

- Risk of the wound infiltration: the local risk may concern the healing and the infectious risk. The local infectious risk does not seem to be increased by wound infiltration when the usual precautions, aseptic technique, are taken (establishment surgery). A meta-analysis to be published, consisting of all the work having put in place a catheter for continuous infusion, shows a significant trend in the reduction of the incidence of infection of the surgical site. The explanation could be on the one hand the direct antiseptic effect of local anesthetics (although the concentrations used), and on the other hand the beneficial effect afforded by the analgesia on microcirculation and local immunity. The risk of poor wound healing is regularly raised but no problem of healing has been reported to date for the hundreds of patients included in the published evaluations (21).

- Risks of the bilateral transverse block of the abdomen: the local anesthetic must be injected into the plane separating the external fascia of the transverse muscle and the internal fascia of the large oblique muscle, or around the branches of the sensory nerves of the abdominal wall; this plane is not easily surgically recognizable and its infiltration by surgical track has not been described. This plane is identifiable from the outside on the inside by the technique of loss of resistance of the fascia (princeps description by McDonnell (19)), a technique which tends to be overshadowed by the ultrasound approach (22, 23). The potential complications of the transverse bilateral block of the abdomen are intra-vascular injection and intra-peritoneal penetration which are exceptional when the appropriate material is used and when the safety tests (suction) are performed, especially as the security of the technique seems to be increased by the echo-guided technique which will be used (22, 23).

**2.5. DESCRIPTION AND JUSTIFICATION OF THE ROUTE OF ADMINISTRATION, THE DOSAGE, THE SCHEMA OF ADMINISTRATION AND THE DURATION OF TREATMENT**

**2.5.1. Description**

The analgesia will compare

- A loco-regional technique

~ Continuous wound infiltration: continuous flow of 5 ml/h levobupivacaine (1.25 mg/ml),

~ or bilateral TAP block: bilateral injection of 20 ml of levobupivacaine (3.75 mg/ml),

- Associated with a systematic analgesic treatment combining for 48 hours:

~ Paracetamol (1 g x 4 per os), nefopam (Acupan, 20 mg x 4 per os), ketoprofen (Profenid, 50 mg x 4 per os)

~ Administration of supplementary Sevredol, 10 mg per os, if the pain score at rest is higher than or equal to 4.

- Administration at the request of patients of paracetamol (1 g x 4 per os) and of nefopam (Acupan, 20 mg x 4 per os), of the 48th hour post-operative at the outlet of the hospital.

**2.5.2. Justification**

These elements are derived from data in the literature:

- For the continuous wound infiltration:

~ Ganta et al. Comparison of the effectiveness of bilateral ilioinguinal nerve block and wound infiltration for postoperative analgesia after cesarean section. Br J Anaesth 1994; 72: 229-30.

~ Mecklem et al. Efficacy of bupivacaine delivered by wound catheter for post-Cesarean section analgesia. Aust N Z J Obstet Gynaecol 1995; 35: 416-21.

~ Fredman et al. The analgesic efficacy of patient-controlled ropivacaine instillation after Cesarean delivery. Anesth Analg 2000; 91: 1436-40.

~ Givens et al. A risk-reduction counseling trial of postoperative wound irrigation with local anesthetic for pain after cesarean delivery. Am J Obstet Gynecol 2002; 186: 1188-91.

~ Lavand'homme et al. Postoperative analgesic effects of continuous wound infiltration with diclofenac after elective cesarean delivery. Anesthesiology 2007; 106: 1220-5.

- For the bilateral TAP block of the abdomen:

~ McDonnell et al. The analgesic efficacy of transversus abdominis plane block after cesarean delivery: a risk-reduction counseling controlled trial. Anesth Analg 2008; 106: 186-91.

**2.6. STATEMENT INDICATING THAT THE RESEARCH WILL BE CONDUCTED IN ACCORDANCE WITH THE PROTOCOL, GOOD CLINICAL PRACTICE AND THE LEGISLATIVE AND REGULATORY PROVISIONS IN FORCE**

The research will be conducted in accordance with the protocol, good clinical practice and the legislative and regulatory provisions in force. The costs of this protocol (drugs and materials) are in charge of the Promoter as well as the expenses related to a possible "pain consultation” (DN4 score greater than or equal to 4). No compensation is provided for the patient.

**2.7. DESCRIPTION OF THE POPULATION A STUDY**

Patients undergoing a cesarean section, performed under spinal anesthesia, are included to participate in the study after their written informed consent.

**2.8. REFERENCES TO THE SCIENTIFIC LITERATURE AND TO THE RELEVANT DATA TO BE USED AS REFERENCE FOR THE SEARCH**

- For the continuous wound infiltration:

~ Mecklem et al. Efficacy of bupivacaine delivered by wound catheter for post-Cesarean section analgesia. Aust N Z J Obstet Gynaecol 1995; 35: 416-21.

~ Fredman et al. The analgesic efficacy of patient-controlled ropivacaine instillation after Cesarean delivery. Anesth Analg 2000; 91: 1436-40.

~ Givens et al. A risk-reduction counseling trial of postoperative wound irrigation with local anesthetic for pain after cesarean delivery. Am J Obstet Gynecol 2002; 186: 1188-91.

~ Lavand'homme et al. Postoperative analgesic effects of continuous wound infiltration with diclofenac after elective cesarean delivery. Anesthesiology 2007; 106: 1220-5.

~ Ganta et al. Comparison of the effectiveness of bilateral ilioinguinal nerve block and wound infiltration for postoperative analgesia after cesarean section. Br J Anaesth 1994; 72: 229-30.

- For the bilateral TAP block of the abdomen:

~ McDonnell et al. The analgesic efficacy of transversus abdominis plane block after cesarean delivery: a risk-reduction counseling controlled trial. Anesth Analg 2008; 106: 186-91.

**3. OBJECTIVES OF THE RESEARCH**

## 3.1. MAIN OBJECTIVE

The main objective of the study was to compare the analgesic efficacy of two techniques of regional analgesia: continuous wound infiltration and bilateral TAP block as a single injection, assuming their equivalence in terms of analgesia.

## 3.2. SECONDARY OBJECTIVE

The secondary outcomes are:

- adverse events of these regional techniques and oral morphine

- patients’ satisfaction,

- peri-cicatricial hyperalgesia (DN4 interview).

**4. RESEARCH DESIGN**

**4.1. SPECIFIC STATEMENT OF THE MAIN CRITERIA OF EVALUATION AND ASSESSMENT OF SECONDARY CRITERIA**

The primary endpoint is the intensity of postoperative pain quantified by the area under the curve (AUC) pain scores (24). Pain was assessed using a numerical rating scale (from 0 corresponding to no pain, to 10 being the worst pain imaginable) at rest and during mobilization (from the supine position in bed to the sitting position). This assessment is performed at H3 (postoperative care unit), H6, H12, H24, H36 and H48. The AUC is calculated by adding the areas under the graph between each pair of consecutive measurements (25). If we have measurements y1, y2 at times t1 and t2, the AUC between these measurements is the product of the time difference and the average of the two measures. Thus, we obtain (t2 - t1) (y1 + y2) / 2. This is known as the “trapezoidal rule” because of the shape of each segment under the curve. If we have n + 1 measurements yi at time t (i = 0,..., n), the AUC is calculated according to the equation:

$$AUC=\frac{1}{2}\sum_{i=0}^{n-1} (t_{i+1}-t_{i})(y_{i}+y_{i+1})$$

Secondary criteria include:

- The delay before the first dose of oral or IV morphine,

- The consumption of oral morphine to H24, H48 and the total dose,

- Adverse effects of regional techniques (non-healing wound infection, puncture of a vessel) and systemic analgesics such as morphine (frequency of pruritus, nausea, vomiting and sedation)

- Resumption of transit,

- The patients’ opinion about analgesia, determined on a numerical rating scale between 1 (very satisfied) and 4 (very dissatisfied)

- Length of hospital stay.

- The occurrence of neuropathic pain assessed by questionnaires DN4: this questionnaire developed by the French Group of Neuropathic Pain, can quickly and simply diagnose a neuropathic pain (26). It includes items obtained during interview (7 items) and clinical examination (3 items). A score of 4/10 correctly identifies neuropathic pain in 86 % of cases, with a sensitivity of 82.9 % and a specificity of 89.9 %. If we use only the interview, a score equal or higher than 4/10 determines positivity of the test (sensitivity 82.9%; specificity of 89.9 %).

**4.2. DESCRIPTION OF THE RESEARCH METHODOLOGY, INCLUDING A SCHEMATIC PRESENTATION WHICH SPECIFIES THE VISITS AND EXAMINATIONS PROVIDED.**

It is a single-center open, randomized, phase 4 study with two groups:

- A group of patients will benefit from continuous wound infiltration,

- The other group will receive a bilateral TAP block.

The study, of which the duration corresponds to the period from the written consent to discharge from hospital, includes the following steps:

- Information about the protocol given during the pre-anesthetic consultation or during the pre-anesthetic visit, the day before the surgery.

- Signature of the consent and inclusion made ​​during the pre-anesthetic visit or ​​on the day of the intervention.

- No premedication.

- Usual monitoring: heart rate, electrocardiogram, pulse oximetry, blood pressure.

- Involving spinal anesthesia with hyperbaric bupivacaine (10 mg if the size is > 1.60 m and 8 mg if height < 1.60 m) and 5 mcg sufentanil.

- Cesarean section.

- Achieving analgesia technique after surgical skin closure.

~ either continuous wound infiltration: insertion of a catheter by the surgeon in the subfascial area, at the top of the incision, connected to an elastomeric pump with 200 ml of levobupivacaine (1.25 mg / ml) providing a continuous flow rate of 5 ml/h of the solution for 40 hours. The total dose of administered levobupivacaine in this group, is 250 mg over 40 hours (6.25 mg/h). The maximal recommended dose with continuous infusion is from 12.5 to 18.75 mg/h (Vidal ™).

~ or bilateral TAP block at the end of the intervention: under ultrasound guidance, a bilateral injection of 20 ml of Levobupivacaine (3.75 mg/ml resulting from the extemporaneous mixing of 10 ml of Levobupivacaine 2.5 mg / ml and 10 ml of levobupivacaine to 5 mg / ml) is performed. The total dose of levobupivacaine administered in this group is 150 mg in a single injection (75 mg for each side). The maximum recommended single dose injection is 150 mg.

- In the postoperative care unit: titration of morphine intravenously if the pain score, at rest, is higher than 4.

- Postoperative administration for 48 hours as routine care of paracetamol (per os 1 g x 4), nefopam (ACUPAN, 20 mg x 4 per os), ketoprofen (PROFENID, 50 mg x 4 per os) and additional administration of oral morphine: SEVREDOL, 10 mg given if the score pain at rest is higher than or equal to 4. A minimum interval of 4 hours is observed between each administration of SEVREDOL.

- Assessment at H3 before leaving the postoperative care unit: numerical pain score, morphine requirement, adverse effects.

- Twice daily monitoring until the 48th postoperative hour with assessment of

~ Numerical Pain score (at rest at bed; at mobilization: from supine position to sitting position on the bed)

~ morphine requirement: Sevredol,

~ Adverse effects: pruritus, nausea, vomiting and sedation, resumption of transit.

- Assessment of the quality of postoperative analgesic prescription during the 48th postoperative hours: paracetamol (1 g x 4 per os) and nefopam (Acupan, 20 mg x 4 per os) if required.

- Visit at the end of the hospital stay with assessment of patient’s satisfaction toward analgesia, determined on a numerical scale from 1 (very satisfied) to 4 (very dissatisfied)

- DN4 interview: phone call at 1 month and eventually proposition of a "specialized consultation for chronic pain" if the established score through the questionnaire was 4 or more.

**4.3. DESCRIPTION OF MEASURES TAKEN IN ORDER TO REDUCE AND AVOID BIAS**

The randomization list is performed by a computer program. It is held by the coordinator of the study.

**4.4. DESCRIPTION OF DOSAGE AND ADMINISTRATION OF THE INVESTIGATIONAL MEDICINAL PRODUCTS. DESCRIPTION OF UNITARY FORM, PACKAGING AND LABELING OF THE INVESTIGATIONAL MEDICINAL PRODUCTS**

Experimental agents are usually available by investigators and are:

- Levobupivacaïne (1.25 mg/ml): continuous wound infiltration

- Levobupivacaïne (2.5 mg/ml and 5 mg/ml): TAP block.

**4.5. EXPECTED PARTICIPATION DURATION OF PATIENTS, CHRONOLOGY DESCRIPTION AND DURATION OF ALL PERIODS OF THE TEST**

For every patient, the duration of the study spreads from the written consent (during the preoperative visit the day before or the day of the surgery) to the third month after cesarean delivery (eventual specialized consultation for chronic and neuropathic pain).

**4.6. DESCRIPTION OF THE RULES FOR FINAL OR TEMPORARY JUDGMENT:**

- For the patient: the patient may decide to withdraw his participation in the study between the time of its signing and the third post-operative month.

- Throughout the research: the research could be interrupted if a severe undesirable effect occurs whatever the cause.

**4.7. PROCEDURES FOR ACCOUNTING OF THE INVESTIGATIONAL MEDICINAL PRODUCTS**

The association of levobupivacaine and ketoprofen is usual. It is found in very many studies, including the major study of Lavand'homme et al. (17).

**4.8. PROVISIONS IMPLEMENTED IN VIEW OF THE MAINTENANCE OF THE KNOWLEDGE AND PROCEDURES OF UNBLINDING, IF APPLICABLE.**

This question does not arise in the context of this research.

**4.9. IDENTIFICATION OF ALL THE DATA COLLECTED DIRECTLY IN THE CRF, WHICH WILL BE CONSIDERED AS SOURCE DATA**

The following tables include the data collected:

| Evaluation for  The eligibility | Data | Formulation |
| --- | --- | --- |
|  | Entry Number | Number |
|  | Date | Date |
|  | Age | Number |
|  | Size (cm) | Number |
|  | Weight (kg) | Number |
|  | ASA Class | Free Text |
|  | History of cesarean section or another abdominal intervention | Free Text |
|  | Preexisting Pathology | Free Text |
|  | Treatment in progress | Free Text |
|  | Comments | Free Text |

| Inclusion | Data | Formulation |
| --- | --- | --- |
|  | Presence of non-inclusion criterion(s) (Yes/No) | Letter |
|  | Refusal to participate (Yes/No) | Letter |
|  | Other reasons for non-inclusion | Free Text |
|  | Inclusion (Yes/No) | Letter |
|  | Comments | Free Text |

| Surgery | Data | Formulation |
| --- | --- | --- |
|  | Start Time | Hour:Minute |
|  | End Time | Hour:Minute |
|  | Surgical Technique | Free Text |
|  | Comments | Free Text |

| Regional Anesthesia | Data | Formulation |
| --- | --- | --- |
|  | Continued Infiltration of the scar (Yes/No) | Letter |
|  | Transverse bilateral block of the abdomen (Yes/No) | Letter |
|  | Problem during puncture | Free Text |

| Data collected in monitoring room post-interventional | Data | Formulation |
| --- | --- | --- |
|  | Titration of morphine (mg) | Number |
|  | Pain Score at rest to H3 | Number |
|  | Pain Score in the effort to H3 | Number |
|  | Comments | Free Text |

| Data collected on the floor of hospitalization | Data | | | Formulation |
| --- | --- | --- | --- | --- |
|  | Pain assessment | | | Number |
|  | At rest | | Evening of D0 | Number |
|  |  | | Morning of D1 | Number |
|  |  | | Evening of D1 | Number |
|  |  | | Morning of D2 | Number |
|  |  | | Evening of D2 | Number |
|  | At mobilization | | Evening of D0 | Number |
|  |  | | Morning of D1 | Number |
|  |  | | Evening of D1 | Number |
|  |  | | Morning of D2 | Number |
|  |  | | Evening of D2 | Number |
|  | Secondary effects : pruritus, nausea, vomiting and sedation | | | Free Text |
|  | Resumption of transit |  | | Free Text |
|  | Overall assessment of analgesia | Day of the output | | Number |
|  | Condition of the scar | Day of the output | | Free Text |
|  | Comments | Day of the output | | Free Text |

| Data collected at the end of in the 1^st^ month | Data | Formulation |
| --- | --- | --- |
|  | Questionnaire DN4 | Number |
| Pain  consultation | Comments |  |

| Exclusion | Data | Formulation |
| --- | --- | --- |
|  | Why | Free Text |

**5. SELECTION AND EXCLUSION OF PATIENTS IN THE RESEARCH**

## 5.1. CRITERIA FOR INCLUSION OF PERSONS THAT ARE SUITABLE FOR THE SEARCH

Patients will be included if:

- a cesarean delivery was scheduled to be performed under spinal anesthesia,

- the height is more than 1.55 m,

- the ASA score is I or II

- the newborn is single,

- whatever their wish of breastfeeding,

- written consent was signed.

## 5.2. CRITERIA OF NON-INCLUSION OF PERSONS THAT ARE SUITABLE FOR THE RESEARCH

Will not be included patients meeting one of the following criteria:

- Less than 18 years old,

- Contra-indications to levobupivacaine: known hypersensitivity to local anesthetics (amide type or to any of the excipients); severe arterial hypotension.

- Contra-indications to paracetamol: allergy, hypersensitivity to acetaminophen or to any excipients, hepatic failure, hepatic porphyria, G6PD deficiency.

- Contra-indications to ketoprofen: hypersensitivity to ketoprofen or any excipients, history of asthma triggered by taking ketoprofen or non-steroid anti-inflammatory agents or aspirin, gastrointestinal hemorrhage, cerebrovascular bleeding or other evolving bleeding, severe hepatic impairment, severe renal impairment, severe uncontrolled heart failure, bleeding disorders or treatment by anticoagulant.

- Contra-indications to nefopam: allergy, hypersensitivity to Nefopam or to any excipients, history of seizures, epilepsy, glaucoma closed angle.

- Contra-indication to morphine per os: hypersensitivity to morphine or one of the excipients, decompensated respiratory failure (without ventilation artificial), severe hepatocellular insufficiency (with encephalopathy), epilepsy, association with buprenorphine, nalbuphine and pentazocine.

**5.3. PROCEDURE ALLOWING PREMATURE DISCONTINUATION OF THE TREATMENT**

**5.3.1. Criteria and modalities of premature discontinuation of treatment or exclusion of a subject from the research**

The treatment will be stopped if the analgesic of choice is deemed to be insufficient by the patient. This leads to the exclusion from the research of the person, who will receive other pain relief treatment.

**5.3.2. Modalities and timetable of compendium for these data**

These data are recorded in real time on an electronic datasheet.

**5.3.3. Arrangements for the replacement of these persons, if applicable**

Patients will be recruited to replace those excluded. The Consort diagram indicates cases where patients are excluded after randomization and start of the treatment. This will be taken into account in the statistical analysis.

**5.3.4. Ways of monitoring these people**

The patients excluded will be followed during anesthesia and in the 3 months following by physicians, anesthesiologists and nurses in the anesthesia department of Hôpital Foch, obstetric management being carried out by the obstetrical team. They will receive care appropriate to their condition.

**5.3.5. Simultaneous Participation in another research, period of exclusion**

It is not necessary to provide for a period during which the person, who participated in this test, will not participate in other research since:

- Levobupivacaine has a short half-life,

- This test includes the same analgesics as those which would have been administered if this person was not involved in the research. In the same way, the person, who participated in this test, could already be participating in other research.

**6. TREATMENT ADMINISTERED TO PERSONS THAT ARE SUITABLE FOR THE RESEARCH**

**6.1 DESCRIPTION OF THE TREATMENTS NECESSARY TO ACHIEVE THE RESEARCH**

The drugs used during the search are

- The levobupivacaine (Chirocaine) administered either by sub-fascial catheter or in the TAP space,

- The bupivacaine and sufentanil administered to achieve the spinal anesthesia,

- Paracetamol, ketoprofen (Profenid) and morphine (Sevredol) as analgesic adjuvants.

**6.2. MEDICINES AND TREATMENTS AUTHORIZED AND PROHIBITED IN THE FRAMEWORK OF THE PROTOCOL, INCLUDING RESCUE Medication**

The nature of a treatment administered during the protocol will be indicated in the specifications of observation.

**6.3. METHODS OF MONITORING THE COMPLIANCE TO TREATMENT**

This question does not arise in this study.

**6.4. STORAGE CONDITIONS OF INVESTIGATIONAL MEDICINAL PRODUCTS**

The drugs will be supplied by the pharmacy of Hôpital Foch.

# 7. ASSESSMENT OF EFFECTIVENESS

## 7.1. DESCRIPTION OF THE PARAMETER FOR THE ASSESSMENT OF EFFECTIVENESS

The main criterion of judgment is the post-operative pain.

**7.2. METHODS AND TIMETABLE SET OUT TO MEASURE, COLLECT, AND ANALYZE THE ASSESSMENT PARAMETER OF THE EFFECTIVENESS**

The main criterion of judgment is measured in real time.

# 8. EVALUATION OF SAFETY

Patient safety is ensured by

- The permanent presence of the anesthesiologist and / or nurse during the peroperative period and by the presence of the anesthesiologist and / or nurse during the post-anesthetic period in postoperative care unit.

- By the nurses of the Department of Obstetrics and Gynecology during the period of

hopital stay after surgery

- The follow-up by an anesthesiologist and / or a nurse from the “pain-unit” after surgery.

**8.1. DESCRIPTION OF THE PARAMETERS OF SAFETY EVALUATION**

These parameters are derived from the monitoring described above.

**8.2. METHODS AND TIMETABLE SET OUT TO MEASURE, COLLECT, AND ANALYZE THE EVALUATION SAFETY PARAMETERS**

These parameters are measured and collected in real time. They are analyzed at the end of each inclusion.

The proponent has found it unnecessary to establish an independent supervisory committee, the data collected having no sensitive nature.

**8.3. PROCEDURES PUT IN PLACE FOR RECORDING AND NOTIFICATION OF ADVERSE EVENTS**

**8.3.1. Definitions**

The investigators use the following definitions:

- Adverse Event: any harmful manifestation occurring in a person who participating in biomedical research, this event being linked or not to the research or to the products used for this research

- Undesirable effect: undesirable effect of the research, any adverse event due to the latter.

- Unexpected Adverse Event: any adverse reaction, whose nature, severity or evolution does not match with the information related to the products, practical acts and methods used in the course of the research.

- Event or serious adverse reaction: any event or undesirable effect which resulted in death, involved the vital prognosis, necessitated the extension of hospitalization, caused a disability or a significant handicap.

**8.3.2. Declaration of serious adverse events**

Any serious adverse event related or non-treatment studied, expected or unexpected, will have to be reported within 24 hours to the proponent on a "serious adverse event" form on which will be indicated the date of occurrence, the intensity, the relationship with the research, and monitoring.

The narrative report should be completed and forwarded to the promoter of the obtaining of relevant new information. Depending on the nature and seriousness of the event, copies of the medical record of the patient can be attached, as well as the results from the laboratory analyzes.

When a serious adverse event is still present at the end of the study, the investigator will follow the patient until the event is considered as resolved.

**8.3.3. Declaration of serious adverse effects**

All suspected unexpected serious adverse reactions will be the subject of a statement of the proponent to the CPP and the French Agency for Food Safety of health products, within the deadlines set.

The detailed records of all adverse events reported by the investigators will be transmitted to the competent authority, at its request.

**8.4. TERMS AND DURATION OF THE FOLLOW-UP OF PERSONS FOLLOWING THE OCCURRENCE OF ADVERSE EVENTS**

The follow-up would be suitable if an undesirable effect proved to be at the origin of a complication requiring treatment or an extension of hospitalization.

**9. STATISTICS**

**9.1. DESCRIPTION OF THE STATISTICAL METHODS, INCLUDING THE CALENDAR OF INTERMEDIATE ANALYSES PLANNED**

Statistical analysis will compare qualitative data (Fischer test) and quantitative data (Mann-Whitney test) of both groups: «continuous wound infiltration» et «bilateral TAP block».

**9.2. EXPECTED NUMBER OF PEOPLE TO INCLUDE IN THE SEARCH, AND EXPECTED NUMBER OF PERSONS IN EACH PLACE OF RESEARCH WITH ITS STATISTICAL JUSTIFICATION**

This equivalence study includes a significance level of 5%. The test has a power of 80% if we consider that the critical difference for the area under the curve during the index period is reached when the ratio between the effects of methods on the primary endpoint is not between 0.8 and 1.25. The coefficient of variation (Standard Deviation/mean) used for the primary endpoint is 0.4. This leads to the need to include two parallel groups of 55 patients and therefore two groups of 60 patients given the risk of attrition.

**9.3. DEGREE OF STATISTICAL SIGNIFICANCE PLANNED**

## The threshold of significance chosen is p < 0.05.

**9.4. STATISTICAL CRITERIA TO STOP THE SEARCH**

No intermediary statistical analysis is planned.

**9.5. METHOD FOR TAKING ACCOUNT OF THE MISSING DATA, UNUSED OR NON-VALID**

The analysis is based on data collected by the investigators and analysis of computer files. Some missing data could be found in the medical files. Missing data due to loss of computer files will cause excluding analysis of patients and their replacement.

The analysis will be carried out by intention to treat.

## 9.6. MANAGEMENT OF CHANGES TO THE PLAN OF ANALYSIS OF THE INITIAL STRATEGY

It is not intended to change the plan of analysis.

**9.7. CHOICE OF PERSONS TO INCLUDE IN THE ANALYSES**

All patients will be analyzed.

**10. ACCESS RIGHT TO DATA AND SOURCE DOCUMENTS**

**10.1. ACCESS TO DATA**

The proponent has obtained the agreement of all the parties involved in the research in order to ensure direct access to all places of conduct of the research, the data source, the source documents and reports in a purpose of quality control and audit by the promoter.

The investigators will make available the documents and individual data strictly necessary for the monitoring, quality control and auditing of the biomedical research, to be at the disposal of persons mandated by the proponent in accordance with the provisions of laws and regulations in force (articles L. 1121-3 and R. 5121-13 of the public health code).

**10.2. SOURCE DATA**

The source documents are defined as any document or original objects allowing proof of the existence or the accuracy of a given or a fact recorded in the course of the clinical trial.

The medical record of each patient will be retained by the health institution where the research is being conducted in accordance with French regulations.

**10.3. CONFIDENTIALITY OF DATA**

In accordance with the provisions concerning the confidentiality of the data to which have access the persons responsible for the quality control of a biomedical research (article L. 1121-3 of the public health code), in accordance with the provisions relating to the confidentiality of information concerning, inter alia, the nature of the investigational medicinal products, the tests, the persons who are appropriate and the results obtained (article R. 5121-13 of the public health code), the persons having a direct access will take all the necessary precautions to ensure the confidentiality of information relating to persons who are loaned to the research, particularly as regards their identity as well as to the results obtained. These people, in the same way as the investigators themselves, are subject to the conditions of professional secrecy (according to the conditions defined by the articles 226-13 and 226-14 of the penal code).

During the biomedical research or to its outcome, the data collected on persons who are suitable and transmitted to the sponsor by the investigators (or all other niche players) will be made anonymous. Patients will be known only by their number of entry in the study and the number of the investigating center. These elements are present in the specification of electronic observation and are attached to the computer files from the monitoring. The collected data are strictly confidential. They are viewed by the medical team, the persons who are duly authorized by the sponsor of the research and possibly by representatives of health authorities and judicial authority. The identity of participants will not be disclosed in any report or publication resulting from this study.

  The proponent will ensure that each person who lends itself to the research has given an agreement in writing for access to the individual data the concerning and strictly necessary to quality control of the research.

**10.4. REGISTRATION IN THE NATIONAL FILE OF PERSONS AGREEING TO A BIOMEDICAL RESEARCH**

This registration is not necessary in the context of research.

**11. MONITORING AND QUALITY ASSURANCE**

**11.1. QUALITY ASSURANCE**

An Independent Clinical Researcher (ICR) mandated by the proponent will ensure the proper conduct of the study, the compendium of data generated in writing, of their documentation, recording and report, in agreement with the Standard Operating Procedures implemented within the Clinical Research Unit of the Foch Hospital and in accordance with Good Clinical Practices as well as the legislative and regulatory provisions in force.

**11.2. QUALITY CONTROL**

The quality-control of the test is carried out under the responsibility of the Clinical Research Unit of Hôpital Foch (Professor Devillier).

**11.3. SPECIFICATION OF OBSERVATION**

All of the information required by the protocol will be reflected in a workbook of electronic observation (ECRF) then posted on the central server of the promoter. The data will be collected as they will be obtained on an electronic database.

**12. ETHICAL CONSIDERATIONS**

The research is based on the latest state of scientific knowledge concerning the analgesia after cesarean section. It includes the administration of the same anesthetic agents that during the clinical usual practice.

No constraint is imposed in this research.

**12.1. Committee for the Protection of Persons**

The protocol, the information form and consent will be submitted for opinion to the Committee for the Protection of Persons Ile de France VIII.

The notification of the favorable opinion of the CPP will be transmitted to the sponsor of the study and to health authorities. An application for authorization will be addressed by the proponent to the French Agency for Health Safety of health products.

# A final report on the research will be directed to the AFSSAPS in the 12 months that follow the end of the study.

**12.2. Information for the patient and informed consent form writing**

Patients will be informed in a comprehensive and fair manner, in understandable terms, of the objectives and constraints of the study, possible risks incurred, measures of surveillance and necessary security, of their rights to refuse to participate in the study or of the possibility of retracting at any time.

All this information is contained on a form of information and consent given to the patient. The free consent, informed and written consent of the patient will be collected by the investigator, or a doctor who represents him, before the final inclusion in the study. A copy of the form of information and consent signed by the two parties will be handed over to the patient, the investigator will retain the original. A copy retained by the Promoter will be placed at the end of study in an envelope sealed tamperproof comprising all of the consent forms.

Any substantial modification of the Protocol, concerning the objectives of the study, its plan, the population, or the administrative significant aspects, will require the approval of the investigator, the sponsor, the favorable opinion of the CPP and the French Agency for Health Product Safety.

# 13. DATA PROCESSING AND RETENTION OF DOCUMENTS AND DATA RELATING TO THE RESEARCH

# 13.1. Entry and processing data

The data for the development of the study are listed in the table below which gives, for each document, its support, its treatment and its contents.

The investigator center has a secure archive, accessible only to the investigators and allowing the local storage of the data of the protocol and the nominative data.

|  | Media | Treatment | Contents |
| --- | --- | --- | --- |
| Form of consent | Paper | Document filled out by the patient and archived by the center investigator in a secure local, accessible only to the investigators. | This document contains the patient’s identity.  It is the only document that allows to find the patient from the entry number.  It contains proof of the consent of the patient and can be used in case of audit. |
| Fact Sheet inclusion | Paper | Document filled out in paper form and archived in the center. | This sheet contains:  - The entry number,  - The clinical information necessary for the realization of the protocol  This sheet contains no reference to the patient’s identity. |
| Tracking Data | Paper  Then Digital | Document filled out in paper form and archived in the center. | These data do not contain the information of the patient’s identity. |

The principal investigator center has modes of computer storage (database), accessible only to the persons named investigators of the study. The latter transfer via a secure network type of Virtual Private Network (VPN) the information collected on the sheet of inclusion and the data collected during the follow-up. None contain nominative data of participants.

**The computerized processing of personal data is consistent with the provisions of Act No. 2004-801 of 6 August 2004 relating to the protection of individuals with regard to the processing of personal data and amending Law No. 78-17 of 6 January 1978, relating to computing, files and freedom. Each participant may exercise his right of access and rectification guaranteed by articles 39 and 40 of the said act, in addressing the**doctor that follows in the context of this research and who knows the patient’s identity.

The statistical analysis will be conducted by Dr. Dreyfus, Clinical Research Unit of the Foch Hospital.

**13.2. CNIL**

A declaration to the CNIL will be performed according to the procedures of the Hôpital Foch.

**13.3. Archive**

The following documents will be archived by the name of the study on the premises assigned to this effect by the sponsor until the end of the period of practical usefulness. These documents are:

- Protocol and Annex, possible amendments,

- Information forms and originals consents signed,

- Copy of computer files corresponding to the individual data,

- Documents of potential monitoring,

- Statistical analyses,

- Final report of the study.

At the end of the period of practical usefulness, the whole set of documents to be archived, as defined in the procedure of "filing and archiving of documents related to biomedical research" of the Hôpital Foch will be transferred to the premises assigned to this effect by the promoter. They will be placed under the responsibility of the promoter for 15 years after the end of the study in accordance with the institutional practices.

No displacement or destruction will be made without the agreement of the promoter. At the end of 15 years, the promoter will be consulted for destruction. All the data, all the documents and reports will be the subject of audit or inspection.

**14. FINANCING AND INSURANCE**

A contract of insurance, in accordance with Article L. 209-7 of the Public Health Code has been purchased from the SHAM, 18 rue Edouard Ratchet, 69372 Lyon Cedex 08 (contract N^o^. 106161 This insurance covers the liability of the promoter and the other stakeholders and may be consulted at the Delegation of the research of the Hôpital Foch.

**15. RULES RELATING TO THE PUBLICATION**

The search will be saved on the site Clinical Trials.

This monocentric study will be the subject of publications in the form of a communication and of an original article. The order of the signatories will be determined by Professor Marc Fischler, principal investigator and coordinator of the study.

**16. LIST OF ANNEXS**

# The following documents are appendices:

- Annex 1: Summary of characteristics of Chirocaine 1.25 mg/ml

- Annex 2: Summary of characteristics of Chirocaine 2.5 mg/ml

- Annex 3: Summary of characteristics of Chirocaine 5 mg/ml

- Annex 4: Questionnaire DN4

- Main References

~ Lavand'homme et al. Postoperative analgesic effects of continuous wound infiltration with diclofenac after elective cesarean delivery. Anesthesiology 2007; 106: 1220-5.

~ McDonnell et al. The analgesic efficacy of transversus abdominis plane block after cesarean delivery: a risk-reduction counseling controlled trial. Anesth Analg 2008; 106: 186-91.

**17. REFERENCES**
